# Combined surgical and endovascular treatment of congenital bilateral femoral arteriovenous fistulas in a child: a case report

**DOI:** 10.3389/fped.2025.1702455

**Published:** 2025-11-14

**Authors:** Jiejun Xia, Liqi Zhang, Kunshan Chen, Zhe Wen, Jianzhong Huang, Haibo Li

**Affiliations:** 1 Department of Interventional Radiology and Vascular Anomalies, Guangzhou Women and Children’s Medical Center, Guangzhou Medical University, Guangzhou, China; 2 Department of Pediatric Surgery, Guangzhou Women and Children’s Medical Center, Guangzhou Medical University, Guangdong Provincial Clinical Research Center for Child Health, Guangzhou, China

**Keywords:** arteriovenous fistula, case report, combined treatment approach, surgical, endovascular treatment

## Abstract

**Background:**

Congenital arteriovenous fistulas (AVFs) are rare, particularly those involving the bilateral femoral arteriovenous system in an infant. This case report details the successful management of this condition using a combined therapeutic approach.

**Case presentation:**

We report the case of a 6-month-old boy who presented with a congenital bilateral femoral arteriovenous fistula, which led to symptoms of fatigue, shortness of breath, and recurrent pneumonia. The patient was successfully treated with a combined therapeutic approach of surgical ligation on the left side and endovascular coil embolization on the right. At the 10-year follow-up, the patient's clinical and echocardiographic status remained normal. The imaging surveillance protocol included regular duplex ultrasound examinations, which confirmed sustained occlusion of both fistulas with no residual dilated veins in the right thigh. The patient remains asymptomatic with no evidence of recurrence.

**Conclusion:**

This case highlights the importance of a tailored, combined-modality approach for complex congenital AVFs, especially when the fistulas on each side possess different anatomical characteristics. The specific morphology of the fistula is a critical determinant in choosing the optimal treatment strategy to ensure complete closure and prevent long-term complications such as high-output cardiac failure. This approach serves as a valuable clinical reference for the management of similar complex vascular anomalies in the pediatric population.

## Background

Infantile congenital arteriovenous fistulas (AVFs) are extremely rare anomalous communications that bypass the capillary beds. While they can occur anywhere (1), the high-flow shunt in such lesions can lead to severe hemodynamic consequences, such as high-output heart failure in infants, making timely and precise management critical (2). Herein, we describe a novel case of a child with this condition involving the bilateral femoral system. This case highlights a successful and unique combined approach, using surgical ligation on one side and transcatheter coil occlusion on the other. It underscores the importance of a tailored, lesion-specific treatment strategy guided by a multidisciplinary team to achieve a favorable long-term outcome.

## Case presentation

**Figure 1:**
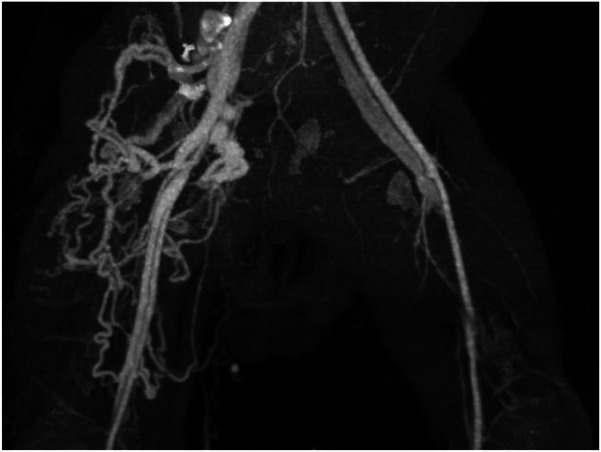
CT angiogram demonstrating bilateral arteriovenous fistulas. The right-sided fistula is supplied by a markedly dilated and tortuous femoral artery and its branches, which drain into a large, tangled venous plexus. This plexus consists of dilated and tortuous femoral and great saphenous veins. In contrast, the left-sided fistula is characterized by moderate dilation of the femoral artery and vein.

A 6-month-old Chinese boy was admitted to our Department of Interventional Radiology and Vascular Anomalies with a one-month history of tachycardia. He was the third child of a healthy, nonconsanguineous couple, born prematurely at 30 weeks gestation via cesarean section, with a birth weight of 1,250 g. There was no known family history of vascular anomalies or related conditions. After 3 months in a neonatal incubator, he was discharged. Two weeks later, the patient presented with symptoms of high-output heart failure, including tachycardia, fatigue, shortness of breath, and recurrent episodes of pneumonia requiring hospitalization. Concurrently, the parents noticed that his right thigh was noticeably thicker than the left, with obvious arterial pulsation. On admission, the boy was in poor condition, presenting with tachycardia (180 bpm; normal range 110–160 bpm) and dyspnea. Physical examination revealed a pulsatile thrill palpable in both inguinal regions and right thigh hypertrophy. Systemic examination revealed no other malformations or dysmorphic features suggesting a genetic syndrome. Given these isolated findings, genetic testing was not performed. An initial chest x-ray showed cardiomegaly, with a cardiothoracic ratio of 0.65, along with dilation of the pulmonary trunk and bilateral pulmonary arteries. An echocardiogram revealed severe pulmonary hypertension (PAH; pulmonary arterial systolic pressure, 65 mmHg) and moderate-to-severe tricuspid regurgitation. Further imaging confirmed direct fistulous tracts communicating the right and left femoral arteriovenous systems (Figure 1).

Given the severity of the patient's symptoms and high-output heart failure, a multidisciplinary team determined that intervention was necessary. A combined therapeutic approach was planned based on diagnostic angiography.

**Figure 2:**
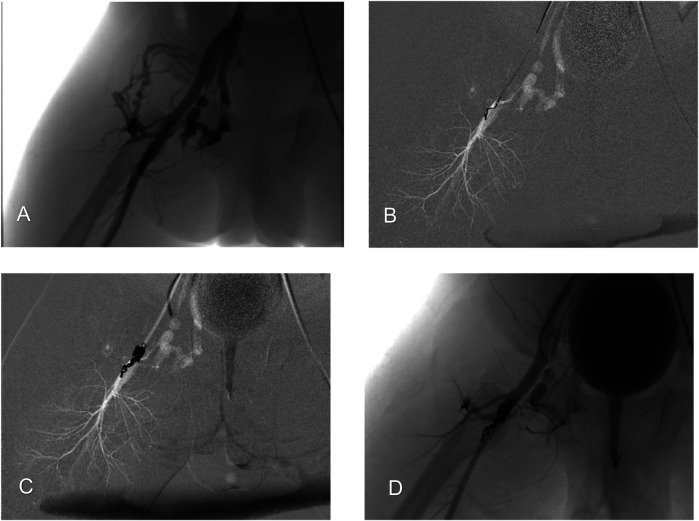
Endovascular embolization of the right-sided arteriovenous fistula. **(A)** Arteriography illustrating a high-flow arteriovenous fistula between the deep femoral artery and femoral vein, with early development of venous backflow. **(B)** A microcatheter was inserted into the fistula orifice for super-selective coil embolization. Coils were anchored to the deep femoral artery and used to progressively fill the fistula. **(C)** Complete occlusion of the fistula orifice was achieved. **(D)** Post-embolization angiography confirms complete occlusion of the fistula orifice, with good visualization of the superficial femoral artery and no signs of distal limb ischemia.

The procedure was performed under general anesthesia. First, endovascular embolization of the right-sided fistula was performed. Angiography via a right femoral artery approach confirmed a complex, multiple arteriovenous fistula with significant ectasia at its origin and venous tortuosity, fed by the deep femoral artery (Figure 2A). A microcatheter was super-selectively advanced into the fistula. Embolization was then performed using 8 Cook spring coils and 2 Interlock detachable coils with fibers (Boston Scientific), which were packed densely to fill the fistula (Figures 2B,C). Post-embolization angiography confirmed complete occlusion of the fistula, with good visualization of the distal superficial femoral artery (Figure 2D).

**Figure 3:**
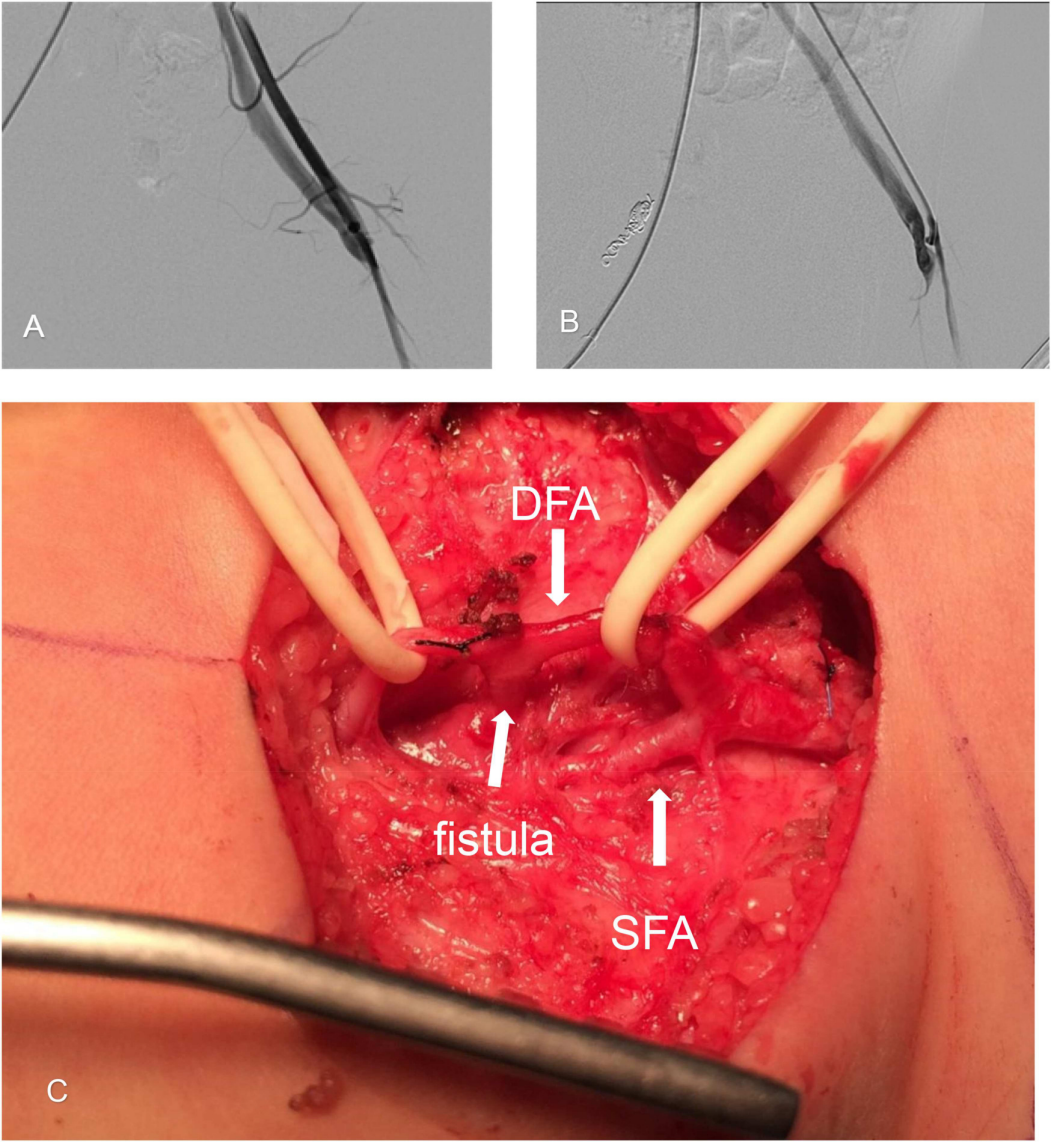
Surgical ligation of the left-sided arteriovenous fistula. **(A)** Arteriography of the left side shows a femoral arteriovenous fistula between the femoral artery and vein. **(B)** Arteriography at the fistula orifice shows abnormal arteriovenous vascular structures filled by feeding arteries arising from the deep femoral artery (DFA). Early venous filling is indicated, demonstrating a short, straight fistula. **(C)** Intraoperative photograph showing an anomalous fistula orifice (indicated by a broad arrow) originating from the deep femoral artery (DFA). The superficial femoral artery (SFA) is also shown.

Next, surgical ligation of the left-sided fistula was performed. Left-sided angiography demonstrated a short, straight, high-flow fistula between the deep femoral artery and vein (Figures 3A,B). A left inguinal incision was made to explore the common femoral artery, superficial femoral artery (SFA), and deep femoral artery (DFA). The anomalous fistula orifice originating from the DFA was identified and ligated (Figure 3C).

The procedures were successful, with no perioperative adverse events recorded. Post-procedure, the patient's pulsatile thrill disappeared, heart rate immediately decreased from 180 bpm to 120 bpm, and dyspnea improved.

At one month post-procedure, a follow-up echocardiogram showed significant clinical and echocardiographic improvement. The ePASP decreased from 65 mmHg to 28 mmHg, and tricuspid regurgitation was reduced from moderate-severe-to mild. The palpable thrill was no longer present.

The patient was subsequently followed in our outpatient clinic. The imaging surveillance protocol consisted of clinical examination and duplex ultrasound of both groins and lower limbs at 6 months, 1 year, and annually thereafter. At the one-year follow-up, the previously noted dilated veins in the right thigh had completely subsided.

**Figure 4:**
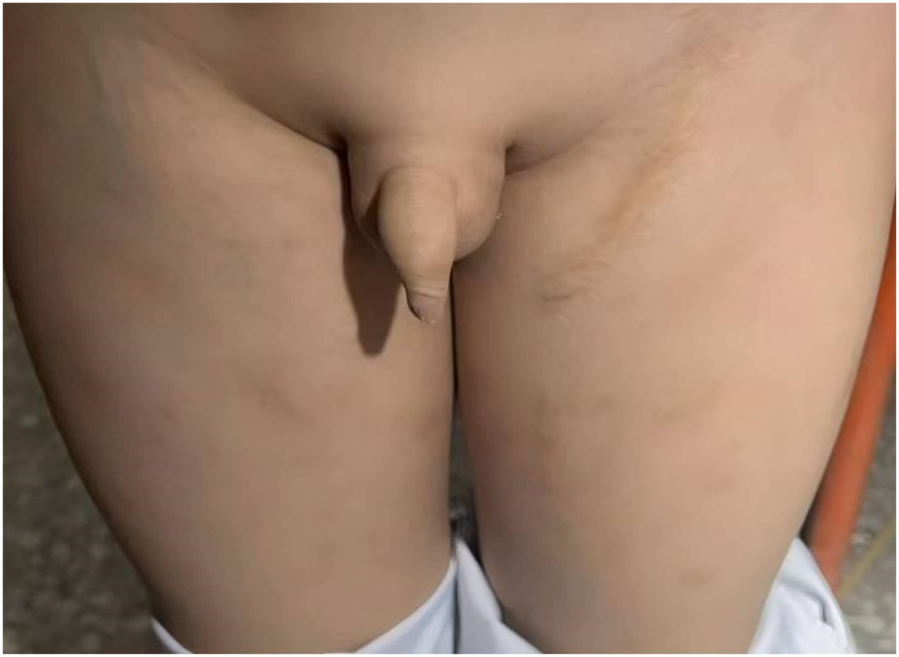
Clinical photographs at the 10-year follow-up. The images demonstrate sustained symmetrical development of both lower extremities. Minor superficial veins are visible on the right thigh, and a well-healed surgical scar is present on the left inguinal region.

At the 10-year follow-up, the patient remained completely asymptomatic with normal lower limb function. Clinical examination confirmed symmetrical development of both lower extremities, with no leg-length discrepancy, edema, or residual hypertrophy of the right thigh, although minor superficial veins were visible on the right side (Figure 4). The surgical scar on the left side was well-healed. Follow-up duplex ultrasound confirmed sustained, stable occlusion of both fistulas, with no signs of recurrence or residual dilated veins. The patient's cardiac status remained normal.

## Discussion

Arteriovenous fistulas (AVFs) are defined as abnormal direct connections between arteries and veins (3). A fundamental concept in the study of vascular anomalies is the critical distinction between an arteriovenous fistula (AVF) and an arteriovenous malformation (AVM) (4). While both are high-flow lesions often broadly but imprecisely referred to as AVMs, their angio-architectural differences are clinically significant. An AVF is a simple lesion characterized by one or a few direct feeding arteries and draining veins, without a nidus. In contrast, a typical AVM consists of a “nidus”—a complex, tangled network of multiple arteriovenous connections that serve as the short-circuit between the arterial and venous systems (5).

An AVF's simple, direct structure creates a low-resistance shunt that can lead to a variety of clinical manifestations, particularly in the pediatric population (6). Local signs in the affected limb often include hypertrophy, skin warmth, a palpable pulsatile thrill, and an audible bruit. A high-flow shunt can cause an increased heart rate, significant cardiac enlargement (cardiomegaly), and ultimately, high-output cardiac failure as a result of increased venous return. As the child grows, these features can become more pronounced, with the potential for developing varicose veins and, in severe untreated cases, tissue ischemia leading to pain, ulceration, and bleeding (2, 7).

The diagnosis of an AVF relies on a comprehensive approach using multiple imaging modalities (8). For our patient, the diagnostic process progressed from initial screening to definitive localization. Ultrasound was key to the initial discovery of the high-flow shunt, while enhanced CT provided a detailed anatomical map of the anomaly and its impact on surrounding structures. However, DSA, the gold standard, precisely delineated the distinct morphologies of the fistulas, serving as the definitive “roadmap” for interventional specialists.

The timing of intervention for pediatric AVFs is a critical consideration. Intervention was primarily driven by the patient's symptomatic high-output heart failure (tachycardia, dyspnea, and cardiomegaly). While asymptomatic, low-flow fistulas might be managed conservatively with close observation, the presence of clear hemodynamic compromise, as seen in our patient, necessitates prompt intervention to prevent progressive and irreversible cardiac damage. Our case thus supports an approach of early intervention in infants presenting with clear clinical and echocardiographic signs of high-output heart failure.

Complete closure of the AVF is the primary treatment goal (9). After angiographically defining the fistula's location and morphology, the choice between surgical and endovascular management is crucial (10). A literature review confirms that congenital femoral AVFs are rare, and bilateral involvement in infancy is especially so. Most reports focus on unilateral lesions, describing successful outcomes with either purely endovascular or purely surgical techniques (11, 12).

Our case contributes to this body of knowledge by presenting a complex bilateral case where a tailored, combined-modality approach, based on the unique angiographic findings of each side, was essential. For the right-sided fistula, which was complex and tortuous with an aneurysmal-like dilation at its origin, endovascular embolization was the ideal choice. Surgical ligation of such a complex fistula would have been challenging and prone to recurrence from collateral channels. As reported by Waigand et al., precise placement of coils within the fistula neck is key (11). This endovascular approach allowed for the super-selective placement of coils, furthermore, the complex anatomy naturally helped to anchor the coil mass, minimizing the risk of migration.

In contrast, the left-sided fistula presented as a short, straight tract with extremely high flow. A transcatheter approach, in this instance, would have carried a high risk of coil migration into the pulmonary circulation, a potentially life-threatening complication. Therefore, open surgical ligation was deemed the safer and more definitive option, providing immediate and complete occlusion. Our lesion-specific, combined strategy proved to be a viable and effective option for this complex, bilateral presentation. Furthermore, the successful 10-year follow-up provides important data on the long-term durability of this combined approach in a growing child, confirming stable occlusion and normal limb development.

## Conclusion

This case report demonstrates the successful application of a multidisciplinary approach for the management of a rare and complex bilateral femoral arteriovenous fistula in an infant presenting with high-output heart failure. Our experience highlights that early diagnosis and timely intervention are crucial in preventing progressive cardiac complications. Furthermore, we show that a tailored, lesion-specific treatment strategy, based on a precise anatomical assessment of each fistula, can lead to excellent long-term outcomes without recurrence. This approach, therefore, serves as a valuable clinical reference for the management of similar complex vascular anomalies in the pediatric population.

## Data Availability

The original contributions presented in the study are included in the article/Supplementary Material, further inquiries can be directed to the corresponding author.
